# Associations of Advanced Glycation End Products with Sleep Disorders in Chinese Adults

**DOI:** 10.3390/nu16193282

**Published:** 2024-09-27

**Authors:** Linyan Li, Jianhe Guo, Xiaoling Liang, Yue Huang, Qiang Wang, Yuxi Luo, Lei King, Liangkai Chen, Xiaolin Peng, Hong Yan, Ruikun He, Jun Wang, Xiaobo Peng, Liegang Liu

**Affiliations:** 1 Department of Nutrition and Food Hygiene, Hubei Key Laboratory of Food Nutrition and Safety, School of Public Health, Tongji Medical College, Huazhong University of Science and Technology, Wuhan 430030, China; d202181656@hust.edu.cn (L.L.); gjhggws123@163.com (J.G.); d202281805@hust.edu.cn (X.L.); d201881303@hust.edu.cn (Y.H.); d201981402@hust.edu.cn (Q.W.); 19871226551@163.com (Y.L.); d202281802@hust.edu.cn (L.K.); clk@hust.edu.cn (L.C.); xiaobopeng92@hust.edu.cn (X.P.); 2 Ministry of Education Key Lab of Environment and Health, School of Public Health, Tongji Medical College, Huazhong University of Science and Technology, Wuhan 430030, China; yanhong@mails.tjmu.edu.cn; 3 Department of Non-Communicable Disease Prevention and Control, Shenzhen Nanshan Center for Chronic Disease Control, Shenzhen 518054, China; xiaolinpeng@hotmail.com; 4 CAS Engineering Laboratory for Nutrition, Shanghai Institute of Nutrition and Health, Chinese Academy of Sciences, Shanghai 200031, China; herk@by-health.com; 5 School of Food and Drug, Shenzhen Polytechnic University, Shenzhen 518055, China; junwangwh@hotmail.com

**Keywords:** advanced glycation end products, Maillard reaction, UPLC-MS/MS, sleep duration, sleep quality, sleep disorders, actigraphy

## Abstract

Background: Advanced glycation end products (AGEs), a group of food processing byproducts, have been implicated in the development of various diseases. However, the relationship between circulating AGEs and sleep disorders remains uncertain. Methods: This cross-sectional study elucidated the association of plasma AGEs with sleep disorders among 1732 Chinese adults who participated in the initial visit (2019–2020) of the Tongji–Shenzhen Cohort (TJSZC). Sleep behavior was assessed using self-reported questionnaires and precise accelerometers. Plasma levels of AGEs, including Nε-(Carboxymethyl)lysine (CML), Nε-(Carboxyethyl)lysine (CEL), and Nδ-(5-hydro-5-methyl-4-imidazolone-2-yl)-ornithine (MG-H1), were quantified by ultra-high performance liquid chromatography–tandem mass spectrometry (UPLC-MS/MS). Results: In logistic regression, per IQR increment in individual AGEs was associated with an increased odds ratio of short sleep duration (CML: 1.11 [1.00, 1.23]; CEL: 1.16, [1.04, 1.30]), poor sleep quality (CML: 1.33 [1.10, 1.60]; CEL: 1.53, [1.17, 2.00]; MG-H1: 1.61 [1.25, 2.07]), excessive daytime sleepiness (CML: 1.33 [1.11, 1.60]; MG-H1: 1.39 [1.09, 1.77]), and insomnia (CML: 1.29 [1.05, 1.59]). Furthermore, in weighted quantile sum regression and Bayesian kernel machine regression analyses, elevated overall exposure levels of plasma AGEs were associated with an increased risk of sleep disorders, including short sleep duration, poor sleep quality, excessive daytime sleepiness, and insomnia, with CML being identified as the leading contributor. Insufficient vegetable intake and higher dietary fat intake was associated with an increase in plasma CEL. Conclusions: These findings support a significant association between plasma AGEs and sleep disorders, indicating that AGEs may adversely influence sleep health and reducing the intake of AGEs may facilitate preventing and ameliorating sleep disorders.

## 1. Introduction

Sleep is intricately involved in numerous physiologic processes and plays a vital role in maintaining physical, social, and mental health. Sleep disorders of different dimensions, such as abnormal sleep duration, decreased sleep quality, excessive daytime sleepiness, and insomnia, have emerged as a prevalent public issue, with the prevalence among Chinese residents reaching 19.16% [1]. Moreover, individuals with sleep disorders exhibit a higher risk of cardiovascular diseases, cognitive decline, and many other medical conditions [2,3,4]. Therefore, there is a pressing need to fully understand the risk factors for sleep disorders, especially modifiable ones like diet, which has great potential for preventing and alleviating sleep disorders.

Previous studies have demonstrated that diet plays a crucial role in regulating sleep. A healthy eating pattern can improve sleep quality, while an unhealthy diet may lead to sleep disorders. Along with lifestyle changes, the consumption of ultra-processed foods (UPFs) has been continuously increasing in recent decades. The growing popularity of UPFs has raised concerns regarding their detrimental impacts on health. Currently, increasing evidence suggests an inverse association between UPFs’ consumption and sleep characteristics, such as sleep duration and quality [5,6,7].

Advanced glycation end products (AGEs), a heterogeneous group of compounds formed through the Maillard reaction, are increasingly recognized as potentially harmful components of UPFs [8]. A growing body of evidence indicates the engagement of AGEs in the pathogenesis of numerous age-related morbidities [8] by inducing oxidative stress, inflammation, and apoptosis [9]. Under normal physiological conditions, endogenous AGEs are slowly produced through nonenzymatic glycation of proteins, lipids, and nucleotides with reducing sugars. This process can be exacerbated by hyperglycemia and oxidative stress [8]. Moreover, consuming AGEs-rich foods, particularly UPFs, introduces exogenous AGEs, which are considered as the primary contributors to the biological AGE pool [9,10,11]. After ingestion, the higher AGE content in UPFs is converted into biological AGEs through gastrointestinal digestion and absorption [12]. Additionally, UPFs generally contain higher levels of sugars, fats, and proteins. As these macronutrients are released during digestion, they facilitate the glycation of proteins in the body [13,14].

AGEs, as potential endocrine-disrupting chemicals, may likely serve as crucial molecules linking UPFs with their detrimental health implications [9,15]. However, to our knowledge, only limited evidence indicates that an increased burden of biological AGEs may lead to decreased sleep quality [16]. Animal studies have observed an enhanced cortisol arousal response [17] and reduced levels of sleep-promoting neurotrophic factor [18] with the increase in circulating AGEs. However, evidence regarding the relationships between plasma AGEs and sleep is lacking. Since diet is the main exogenous source, exposure to the mixture of AGEs that are ubiquitous in the diet is inevitable. In addition, AGEs accumulate gradually in the body over time, which makes humans more prone to aging and chronic diseases [8,9,19,20]. Therefore, understanding the dietary factors associated with plasma AGE levels is conducive to proposing effective dietary interventions to reduce dietary AGEs intake.

Accordingly, the primary objective of this community-based study was to explore whether plasma AGEs are associated with sleep disorders in Chinese adults. Moreover, considering the nature of simultaneous exposure to multiple AGEs, we investigated the joint association of three typical AGEs with sleep disorders. As a secondary objective, we aimed to investigate the relationship between the nutritional composition of diet and plasma AGEs.

## 2. Materials and Methods

### 2.1. Study Design and Population

Our analysis was conducted using the 2019–2020 baseline survey data of the Tongji–Shenzhen Cohort (TJSZC), the details of which have been reported elsewhere [21]. All participants provided written informed consent. The study was approved by the Ethic and Human Subject Committee of Tongji Medical College, Huazhong University of Science and Technology. This study is in line with the Declaration of Helsinki.

During 2019–2020, a total of 2103 individuals completed the baseline survey. We initially included participants aged 18–80 years, who had complete demographic data and information on sleep duration (*n* = 1968). Subjects with severe diseases (*n* = 113) or insufficient plasma samples (*n* = 115) and pregnant or lactating women (*n* = 8) were then excluded. Finally, 1732 individuals were included in the final analysis. Of these, 383 subjects further reported sleep quality and daytime sleepiness, and a subset of 182 participants who provided wrist actigraphy data for at least 3 nights were included in the analysis of subjective sleep variables (Figure S1).

### 2.2. Plasma AGEs Quantification

The determination of free AGEs in plasma was carried out using a UPLC-MS/MS system consisting of an Agilent 1200SL UPLC system and an Agilent 6460 triple series quadrupole mass spectrometer (Agilent Technologies Inc., Santa Clara, CA, USA). A previous study has provided a detailed description [22]. Briefly, after being mixed with internal standards, the plasma was deproteinized with an acetonitrile methanol mixture (1:3) and centrifuged at 15,000 rpm for 20 min at room temperature. The supernatant was evaporated to dryness under nitrogen, and then re-dissolved in 5 mmol/L nonafluoropentanoic acid. A ZORBAX Eclipse Plus C18 Chromatographic column (Agilent Technologies Inc., Santa Clara, CA, USA) with a mobile-phase gradient elution of acetonitrile and 5 mmol/L sepfluvalerate aqueous solution was employed for chromatographic separation. Three widely characterized AGEs, namely Nε-(Carboxymethyl)lysine (CML), Nε-(Carboxyethyl)lysine (CEL), and Nδ-(5-hydro-5-methyl-4-imidazolone-2-yl)-ornithine (MG-H1) were quantified as representatives of lysine- and arginine-derived AGEs. CML-d2, CEL-d4, and MG-H1-d3 (Iris Biotech GmbH, Marktredwitz, Germany) were used as internal standards for the quantification of CML, CEL, and MG-H1, respectively. The limits of quantification (LOQs) of CML, CEL, and MG-H1 were 0.5, 0.05, and 0.25 μg/L, respectively, and all the inter-assay coefficients of variation and accuracy were within 15%. Detailed parameters are presented in Table S1.

### 2.3. Subjective Sleep Assessment

Subjective sleep disorders, including short sleep duration, poor sleep quality, excessive daytime sleepiness, and insomnia were evaluated through self-administrated questionnaires. Sleep duration (in minutes) was defined as the interval between bedtime and wake time after subtracting the time taken to fall asleep. In accordance with international consensus guidelines [23], sleeping less than 7 h per night was classified as short sleep duration. Sleep quality was evaluated using the Pittsburgh Sleep Quality Index (PSQI) [24], with a score above 5 indicating poor sleep quality [25]. Daytime sleepiness was assessed using the Epworth Sleepiness Scale (ESS) [26], and a score above 9 indicates excessive daytime sleepiness [27]. Insomnia was categorized based on self-reported frequent insomnia symptoms and previous physician diagnoses [28].

### 2.4. Objective Sleep Assessment

The actigraphy sensor (wGT3X-BT, ActiGraph Corp, Pensacola, FL, USA) was worn on the non-dominant wrist for at least three consecutive days while completing a sleep diary to document their nightly bedtime and wake time [29]. The collected data were then analyzed using ActiLife software (version 6.13.4, ActiGraph Corp, Pensacola, FL, USA).

### 2.5. Assessment of Covariates

A directed acyclic graph (DAG) was drawn to select a minimal set of potential covariates for studying the link between AGE levels and sleep health (Figure S2) [30]. Demographic and lifestyle data were collected through standardized questionnaires. Current smoker was defined as smoking more than one cigarette per day, and current drinker was defined as drinking more than once in the past month. Regular physical activity was dichotomized based on self-reported regular exercise habits. The estimated glomerular filtration rate (eGFR) was calculated from serum creatinine concentration (in mg/dL) using the modified Modification of Diet in Renal Disease (MDRD) equation [31]. Dietary habits of individuals were assessed by a questionnaire on the frequency and amount of per intake of vegetable and animal food, then defined as insufficient vegetable intake (<300 g/d) and high animal food intake (>200 g/d) in accordance with the recommended intake in the Dietary Guidelines [32]. For participants with actigraphy data, dietary intake data were collected by a trained investigator using a three-day 24 h dietary record survey, along with questions about frequency of tea and coffee consumption. The macronutrient composition was then calculated using the updated “Chinese Food Composition Tables” [33]. Given the low proportion of coffee consumption, a tea/coffee consumer was defined as drinking tea or coffee at least three times per week.

### 2.6. Statistical Analyses

General characteristics were summarized as number (percentage), mean (standard deviation, SD), or median (interquartile range, IQR) for categorical and continuous variables, respectively. Correlations were evaluated with Spearman rank coefficients. Intergroup comparisons were performed by Wilcoxon rank sum test and Chi-square test. Missing values of covariates were imputed using the “mice” package of R (version 4.4.1).

Initially, single-exposure analyses were executed, such as logistic regression, quantile regression, and restricted cubic spline (RCS). Multivariable logistic regression was employed to investigate the relationships of individual AGEs standardized by the IQR with sleep disorders (as categorical variables). The potential non-linear relationships of Ln-transformed AGEs with sleep disorders were elucidated through RCS. Stratified analyses were conducted to assess the reliability of significant findings from the logistic regression. Since there are no specific assumptions about the residual distribution, quantile regression enables the exploration of associations beyond the mean of outcomes [34]. In this study, quantile regression models were fitted at the 0.25, 0.5, and 0.75 percentiles as complementary analyses to investigate the heterogeneous association between per IQR increments in AGEs and continuous sleep variables.

Subsequently, the weighted quantile sum (WQS) regression model was employed to assess the relationship between the AGEs’ mixture and subjective sleep disorders, with the formula outlined in the prior literature [35] Bootstrapping was conducted with 10,000 iterations in both positive and negative directions. The dataset was randomly split into two subsets: with 40% allocated to the training set and the remaining 60% to the validation set.

Furthermore, Bayesian kernel machine regression (BKMR) was utilized to detect intricate non-linearity and nonadditive correlations among exposures (lnCML, lnCEL, and lnMG-H1) and to visualize the individual exposure–response association [36,37]. Previous studies have outlined the application of probit BKMR for binary outcomes and BKMR for continuous variables [37,38]. A hierarchical variable selection approach, employing the Markov chain Monte Carlo algorithm with 10,000 iterations, was implemented. The impact on sleep was assessed by comparing alterations in response variables when all AGEs were fixed at a certain percentile compared to the 50th percentile [39]. The posterior inclusion probability was calculated to determine the significance of each exposure with a threshold of 0.5 [40]. Univariate dose–response relationships for individual AGEs were examined by keeping other AGEs at their 25th, 50th, and 75th percentiles [37]. Bivariate dose–response functions were then utilized to explore potential interaction effects [21].

All statistical analyses were conducted using SAS (version 9.4, SAS Institute, Cary, NC, USA) and R (version 4.4.1, RStudio, Boston, MA, USA). A two-tailed *p* < 0.05 was considered statistically significant.

## 3. Results

### 3.1. Participants’ Characteristics

The demographic characteristics of the 1732 participants, with a mean age of 51.91 years, are presented in Table 1. The mean self-reported nighttime sleep duration was 425 (390, 470) minutes. The distribution of AGEs (median of CML, CEL, and MG-H1: 25.76, 13.53, and 79.59 μg/L, respectively) is detailed in Table S2. Positive correlations were found between CML and MG-H1 (r = 0.51), CML and CEL (r = 0.18), and CEL and MG-H1 (r = 0.10) (Figure S3).

### 3.2. Associations of Individual AGEs and Self-Reported Sleep Assessments

**Figure 1:**
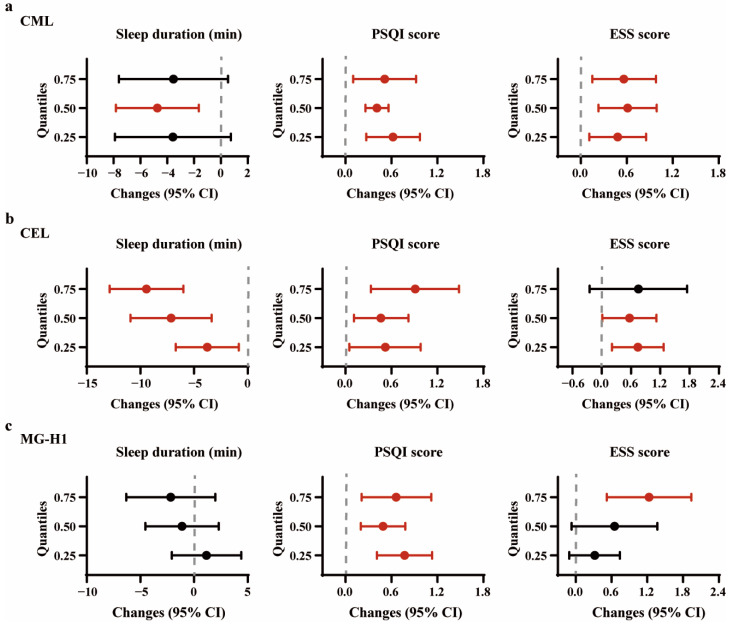
Associations between individual plasma AGEs and various quantiles of subjective sleep variables. (**a**) per IQR increment in CML; (**b**) per IQR increment in CEL; (**c**) per IQR increment in MG-H1. The x-axes depict the changes in subjective continuous sleep variables for an IQR increment in exposure, while the error bars represent 95% confidence intervals. Significant estimated changes (95%CI) are in red. The models were adjusted for covariates including age, sex, BMI, fasting glucose, eGFR, current smoking status, current drinking status, regular physical activity, high animal food intake, and insufficient vegetable intake. Abbreviations: CML, Nε-(Carboxymethyl)lysine; CEL, Nε-(Carboxyethyl)lysine; MG-H1, Nδ-(5-hydro-5-methyl-4-imidazolone-2-yl)-ornithine.

After multivariable adjustment, each IQR increment in plasma CML was linked to an elevated odds of short sleep duration (1.11 (1.00, 1.23)), poor sleep quality (1.33 (1.10, 1.60)), excessive daytime sleepiness (1.33 (1.11, 1.60)), and insomnia (1.29 (1.05, 1.59)) (Table 2). Furthermore, no non-linear relationships were observed in RCS analysis (Figure S4). In quantile regression, each IQR increment in plasma CML levels was found to be associated with a reduction in sleep duration at the median tail (50th percentile), and an increase in the PSQI and ESS score across different percentiles (Figure 1a, Table S3).

Each IQR increment in CEL was positively associated with short sleep duration, and poor sleep quality (OR (95% CI): 1.16 (1.04, 1.30) and 1.53 (1.17, 2.00), respectively) (Table 2). Non-linear relationships were noted for short sleep duration (*p* for non-linear = 0.009, Figure S4). With each IQR increment in CEL, quantile regression analysis demonstrated a significant decrease in sleep duration and increase in PSQI score across various percentiles. Heterogeneous associations were observed in the ESS score, with significance disappearing at the 75th percentile (Figure 1b, Table S3).

For each IQR increment in MG-H1 levels, the odds of participants experiencing poor sleep quality and excessive daytime sleepiness increased by 0.61 (95% CI: 1.25, 2.07) and 0.39 times (95% CI: 1.09, 1.77), respectively (Table 2). RCS analysis further demonstrated a non-linear relationship of sleep duration (Figure S4). In quantile regression, MG-H1 exhibited a positive association with various percentiles of PSQI score, with a significant association observed only at the higher tail (75th percentile) of the ESS score (Figure 1c, Table S3). Notably, the relationships between individual AGEs and subjective sleep disorders remained largely consistent across most subgroups (Figure S5).

### 3.3. Joint Associations in the WQS Analysis

In the WQS regression model, the WQS index was positively associated with increased risks of poor sleep quality, excessive daytime sleepiness, and insomnia (OR (95% CI): 1.57 (1.15, 2.15), 1.69 (1.13, 2.53), and 1.93 (1.05, 3.52), respectively). Among three AGEs, CML demonstrated the highest weight for the aforementioned relationships of poor sleep quality (53.26%), excessive daytime sleepiness (91.95%), and insomnia (61.41%) (Table 3 and Table S4).

### 3.4. Joint Associations in the BKMR Analysis

**Figure 2:**
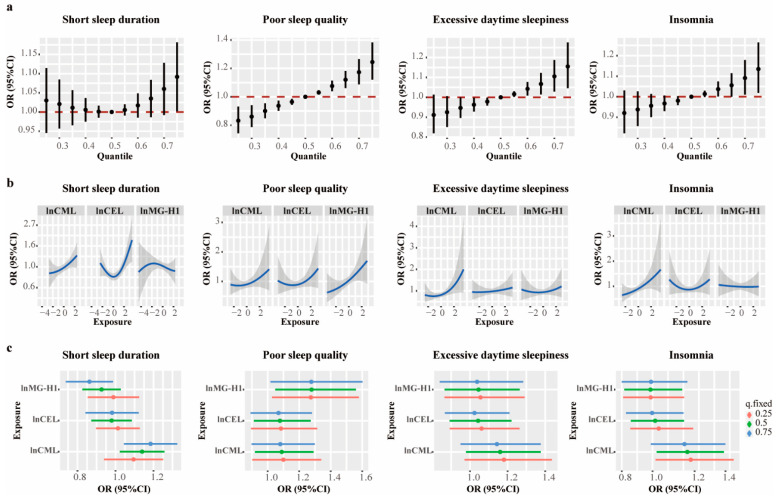
The estimated joint associations of the AGEs’ mixture with sleep disorders in the hierarchical BKMR analysis. (**a**) The overall associations of the AGEs’ mixture with short sleep duration, poor sleep quality, and excessive daytime sleepiness at AGE mixture concentrations ranging from 25th to 75th percentiles relative to the median (50th percentile), black dot represents the estimated OR and error bar indicates 95%CI. (**b**) Univariate exposure–response functions for individual AGEs when the other two were fixed at their 50th percentiles, blue curb line represents the estimated OR and the shaded area indicates 95%CI. (**c**) Estimated OR (95% CI) of risk of short sleep duration, poor sleep quality, and excessive daytime sleepiness for individual AGEs by IQR increment (75th vs. 25th percentile) when the other two were fixed at the 25th, 50th, and 75th percentiles in single exposure–response functions. The models were adjusted for age, sex, BMI, fasting glucose, eGFR, current smoking status, current drinking status, regular physical activity, high animal food intake, and insufficient vegetable intake.

In the BKMR analysis, a higher mixture of AGEs was associated with an increased risk of short sleep duration, poor sleep quality, excessive daytime sleepiness, and insomnia (Table S5). As shown in Figure 2a, compared to individuals at the 50th percentile of the AGEs’ mixture, those at the 75th percentile exhibited an elevated risk of poor sleep quality, excessive daytime sleepiness, and insomnia. While the confidence interval for short sleep duration was broad, borderline statistical significance was observed. The univariate exposure–response function revealed increased associations of CML with short sleep duration, poor sleep quality, and excessive daytime sleepiness when other AGEs were at median levels. (Figure 2b). Moreover, when the other AGEs were set at the 50th and 75th percentiles, CML showed a positive association with short sleep duration (Figure 2c). Additionally, CML was positively associated with insomnia when the other AGEs were set at the 25th and 50th percentiles (Figure 2c). Notably, no interactions were observed among the three AGEs (Figure S6).

### 3.5. Associations of AGEs and Actigraphy-Measured Sleep Variables

We further analyzed 24 h actigraphy data from a subset of 182 participants over a minimum of three consecutive nights (average 4 nights) (Table S6). Similarly, each IQR increment in CML, CEL, and MG-H1 was inversely associated with both total sleep time and sleep efficiency (Figure S7). Additionally, the average WASO was positively associated with an IQR increase in CML and CEL (Figure S7). These associations varied across quantiles (Figure S7). Moreover, in the BKMR models, the AGEs’ mixture was inversely associated with both total sleep time (−10.55, 95% CI: −18.49, −2.61) and sleep efficiency (−1.50, 95% CI: −2.44, −0.55) as measured by actigraphy (Figure S8).

### 3.6. Associations of Dietary Intake and Plasma AGEs

As shown in Table S7, individuals with insufficient vegetable intake had higher plasma CEL levels, but no significant differences in plasma CML and MG-H1 levels. In comparison to individuals with optimal animal food intake, plasma AGEs were lower in those with high animal food intake (Table S7). Spearman correlation analysis of dietary records revealed that higher daily fat intake was linked to increased plasma CEL levels (Table S8). No significant association was observed between dietary vegetable and animal food intake and sleep disorders (Table S9).

## 4. Discussion

This research conducted a comprehensive investigation of the individual and joint relationships of three plasma AGEs with sleep health. Single-exposure models revealed that levels of CML, CEL, and MG-H1 were positively associated with various subtypes of sleep disorders. Furthermore, the WQS models illustrated that AGEs’ mixtures were associated with short sleep duration, poor sleep quality, excessive daytime sleepiness, and insomnia, with CML being the primary contributor. These findings remained robust in the BKMR model. Moreover, the data collected from accelerometers provided further more objective evidence. Largely consistent findings across various statistical methods suggested that AGEs may be factors affecting sleep health.

Previous studies have shown negative associations between high-AGE diets and sleep duration and sleep quality [5,6,7]. In contrast, individuals with high adherence to a low-AGE diet were more likely to report optimal sleep duration and fewer sleep-related problems [41]. However, there is limited evidence on the association between plasma AGEs and sleep. Currently, dietary AGE intake estimates are constrained by incomplete food AGE databases, inconsistent detection methods of foods, and the lack of food preparation details, making it difficult for comparisons between studies [11,42]. Conversely, plasma AGEs, influenced by diet, endogenous production, and metabolic clearance [43], directly reflect the current biological burden of AGEs and are more closely linked to diseases [44,45]. The precise measurement of plasma AGEs avoids recall bias from dietary surveys, making them widely used biomarkers in epidemiological research [46]. Given that dietary AGEs were positively correlated with free AGEs but not protein-bound AGEs in plasma [47,48], we explored the association between plasma-free AGEs and sleep disorders. A significant association between plasma AGEs and sleep disorders was found in this study, which complements the deleterious effects of AGEs on a wide range of physical and mental health issues [9,17,49].

In this study, the results of single-exposure models identified significant adverse associations between free AGEs and both sleep duration and quality, and that individuals with higher AGEs may have more severe daytime sleepiness. However, to the best of our knowledge, only a tiny amount of research has evaluated the links between AGEs and sleep health. A previous cross-sectional study documented a significant increase in circulating CML in women with sleep-discontinuous breathing [50]. Notably, humans are inevitably exposed to complex mixtures of AGEs in their daily lives. The unique health implications of that may be overlooked in single-exposure analysis, as there are significant correlations between AGEs [51]. Thus, we constructed WQS and BKMR models and elucidated that concurrent exposure to elevated AGEs was associated with sleep disorders. Both methods were widely employed for estimating the comprehensive relationships between exposure mixtures and health outcomes.

A previous investigation reported a higher concentration of plasma-free CML of individuals with diabetic nephropathy (46.5 μg/L) compared to our participants (25.61 μg/L) [52]. Chronic hyperglycemia promotes an increased endogenous production of AGEs as a result of the reaction between blood sugars and proteins [53], this may account for the discrepancy in CML levels. Another possible explanation lies in the impaired renal function in diabetic nephropathy, which hinders the clearance of AGEs that relies on urine excretion [12]. Although this study population consisted of healthy adults with normal blood glucose and renal function, considering that circulating AGEs levels are influenced by blood glucose and eGFR [54], we took both as covariates in all analyses to mitigate potential confounding impacts. Conversely, the plasma levels of AGEs in our study (CML: 25.76, CEL: 13.53, MG-H1: 79.59 μg/L) were slightly higher than those of healthy individuals from the Netherlands (CML: 15.6, CEL: 9.7, MG-H1: 25.1 μg/L) [55] and France (CML: 12.5, CEL: 12.7 μg/L) [56]. The difference in AGE levels between the two studies could be explained by age (64.7 ± 8.3 [55] vs. 18–30 years [56]), as it is widely acknowledged that the accumulation of AGEs increases with age. Notably, the participants in our study were younger (mean age 51.91 years) than in the previously mentioned study conducted in the Netherlands [55] but had higher AGE levels, possibly due to regional dietary differences. Thus, the health implications of prolonged AGE exposure deserve more attention.

Diet serves as a major exogenous source of biological AGEs. Shifts in dietary habits and progress in food processing technologies have led to an inevitable and extensive exposure to AGEs, underscoring the necessity of identifying dietary factors that influence the biological burden of AGEs. This study found that individuals with insufficient vegetable intake exhibited elevated plasma CEL levels. This observation aligns with the current understanding that vegetables, which are low in AGEs and rich in antioxidants, contribute to the reduction in AGEs’ formation or accumulation [42,57]. Unexpectedly, we found that plasma CEL levels were lower in individuals with high animal food intake, indicating that cooking methods and certain nutrients (such as protein and fat) in animal foods are more conducive to AGEs’ accumulation [9,11,19]. A positive correlation between daily fat intake and plasma CEL levels supports this notion. Further studies are required to uncover the specific mechanisms by which plant-based and animal food-based diets affect plasma AGEs. Nevertheless, no association was observed between dietary habits and plasma levels of CML and MG-H1. More evidence is warranted to shed light on the health implications of dietary AGEs, as well as elucidate how the regulation of plasma AGEs through the consumption of high-AGE foods like UPFs impacts sleep. This would provide compelling support for the hypothesis that circulating free AGEs may serve as a critical link between UPFs and their adverse health effects [9,15].

Despite the precise mechanisms through which AGEs may adversely affect sleep remaining largely unknown, existing evidence supports several potential avenues. First, higher AGEs are positively related to the cortisol awakening response [17]. Second, CML treatment results in a reduction in tryptophan [18], the raw material for melatonin synthesis. Moreover, it is posited that the disruption of the circadian clock may serve as an additional mechanism, as suggested by the evidence related to acrylamide [58]. Both the secretion of melatonin and the function of the circadian clock are integral in regulating sleep [59,60]. Additionally, the accumulation of AGEs can provoke oxidative stress and inflammation, both of which are potential pathological changes in the development of sleep disorders. In turn, sleep deprivation may also amplify the engagement of neural circuits linked to food stimulation-related reward, pleasure, and salience, resulting in elevated circulating AGEs due to increased consumption of high-fat and high-carbohydrate foods, and UPFs [61].

Our study, with several strengths, thoroughly investigated the individual and joint relationships between plasma AGEs and sleep among Chinese adults. The consistency of results yielded by diverse statistical models enhances the reliability of our findings. Furthermore, we utilized the UPLC-MS/MS method for precise and sensitive measurements of three specific AGEs. It is worth noting that in a subset of participants wearing an actigraphy sensor, more detailed objective sleep indicators were also significantly associated with plasma AGEs, despite the small sample size.

However, several limitations warrant consideration. The cross-sectional design prevented the establishment of causal relationships. This study initially indicated associations between elevated levels of AGEs and poor sleep, but it is also possible that poor sleep could affect food choices [61], leading to higher AGE levels and creating a vicious cycle. Hence, caution is warranted when drawing conclusions and more in-depth research is needed to clarify the bidirectional relationship and underlying mechanisms. Moreover, the limited dietary data in this study precluded the calculation of dietary AGEs’ intake, making it impossible to clearly distinguish the specific dietary sources of plasma AGEs and the contribution of various foods. Therefore, further studies evaluating both dietary and plasma AGEs simultaneously are required to better understand how the regulation of dietary intake on plasma AGE levels affects sleep. Despite the consistency of the findings following adjustments for potential confounders, the possibility of residual and unobserved confounding, such as taking sleeping medications, cannot be entirely ruled out due to the lack of relevant information in the TJSZC. Additionally, with over forty various AGEs identified [19], it is intriguing to investigate the relationships between other AGEs and sleep. Meanwhile, in the assessment of sleep health, the best practice is to incorporate both subjective sleep measures and objective sleep measures obtained from actigraphy or polysomnography. Additionally, although the underlying molecular mechanisms were preliminarily discussed, this study was unable to provide direct evidence but presents the necessity for continued research to understand the mechanisms by which AGEs may cause sleep disorders.

## 5. Conclusions

This study indicated positive associations between levels of AGEs in plasma and sleep disorders in various exposure models. Implementing strategies to reduce AGEs, such as adopting a nutritious and healthy diet, may be beneficial to healthy sleep. However, longitudinal studies and randomized controlled trials are warranted to clarify the causal relationships between AGEs and sleep disorders. The underlying biological mechanisms warrant further investigation.

## Figures and Tables

**Table 1:** Demographic characteristics of participants in this study.

Characteristics	All Participants
Age, years	51.91 (11.55)
Female, *n* (%)	999 (57.68)
Ethnicity/Han, *n* (%)	1697 (97.98)
Body mass index, kg/m^2^	24.55 (3.16)
Fasting blood glucose, mmol/L	5.10 (4.71, 5.75)
eGFR, mL/min/1.73 m^2^	113.55 (99.04, 132.71)
Current smoker, *n* (%)	261 (15.07)
Current drinker, *n* (%)	695 (40.13)
High animal food intake, *n* (%)	385 (22.23)
Insufficient vegetable intake, *n* (%)	1193 (68.88)
Regular physical activity, *n* (%)	1262 (72.86)
Morbidity burden, *n* (%)	
None (0)	629 (36.32)
Moderate (1–3)	1033(59.99)
Significant (4+)	64 (3.70)
Self-reported sleep characteristics ^a^	
Sleep duration, min	425 (390, 470)
Categorical sleep duration, *n* (%)	
Short sleep duration (<7 h)	713 (41.17)
Normal sleep duration (≥7 h)	1019 (58.83)
PSQI score	5 (4, 8)
Categorical PSQI, *n* (%)	
Normal (0–5)	193 (50.39)
Poor sleep quality (>5)	190 (49.61)
Insomnia, *n* (%)	50 (13.06)
ESS score	5 (2, 8)
Categorical ESS, *n* (%)	
Normal/borderline (0–9)	311 (81.20)
Excessive daytime sleepiness (>9)	72 (18.80)
Objective actigraphy-measured sleep characteristics ^b^	
No. nights per participants	4 (3, 6)
Total sleep time, min	360.95 (333.33, 394.00)
Sleep efficiency, %	81.74 (77.04, 86.24)
Wake after sleep onset, min	73.94 (56.63, 96.25)
Energy, kcal/day ^b^	1468.40 (1209.80, 1756.30)
Protein, g/day ^b^	48.76 (38.10, 59.05)
Fat, g/day ^b^	51.74 (41.50, 64.97)
Carbohydrate, g/day ^b^	197.66 (160.22, 241.38)
Tea/coffee consumer ^b^	70 (38.46)

^a^ Subjective sleep duration *n* = 1732; PSQI score, ESS score, and insomnia *n* = 383. ^b^ Actigraphy-measured sleep variables, dietary intake, and tea/coffee consumer *n* = 182. Data were presented as *n* (%) for categorical data, mean (standard deviation), and median (interquartile range) for normally and non-normally distributed data, respectively.

**Table 2:** Associations of per IQR increment of individual plasma AGEs with subjective sleep disorders.

	Short Sleep Duration (<7 h)		Poor Sleep Quality (PSQI >5)		Excessive Daytime Sleepiness (ESS > 9)		Insomnia	
	OR (95% CI)	*p*-Value	OR (95% CI)	*p*-Value	OR (95% CI)	*p*-Value	OR (95% CI)	*p*-Value
CML								
Crude model	1.12 (1.01, 1.23)	0.027	1.36 (1.13, 1.64)	0.001	1.34 (1.12, 1.59)	<0.001	1.35 (1.12, 1.63)	0.001
Model 1	1.11 (1.01, 1.23)	0.031	1.33 (1.10, 1.60)	0.003	1.32 (1.11, 1.58)	0.002	1.32 (1.09, 1.61)	0.005
Model 2	1.11 (1.00, 1.22)	0.046	1.34 (1.10, 1.61)	0.003	1.33 (1.11, 1.59)	0.002	1.30 (1.06, 1.59)	0.014
Model 3	1.11 (1.00, 1.23)	0.044	1.33 (1.10, 1.60)	0.004	1.33 (1.11, 1.60)	0.002	1.29 (1.05, 1.59)	0.017
CEL								
Crude model	1.17 (1.06, 1.30)	0.003	1.55 (1.20, 2.00)	0.001	1.26 (0.95, 1.68)	0.107	1.33 (0.96, 1.83)	0.084
Model 1	1.11 (1.00, 1.24)	0.052	1.50 (1.16, 1.94)	0.002	1.23 (0.91, 1.65)	0.176	1.24 (0.88, 1.75)	0.211
Model 2	1.16 (1.03, 1.30)	0.012	1.52 (1.17, 1.98)	0.004	1.24 (0.92, 1.67)	0.156	1.24 (0.87, 1.76)	0.238
Model 3	1.16 (1.04, 1.30)	0.001	1.53 (1.17, 2.00)	0.002	1.23 (0.91, 1.67)	0.180	1.22 (0.86, 1.74)	0.271
MG-H1								
Crude model	1.04 (0.95, 1.15)	0.395	1.64 (1.28, 2.11)	<0.001	1.35 (1.07, 1.71)	0.011	1.27 (0.98, 1.83)	0.072
Model 1	1.03 (0.93, 1.13)	0.563	1.61 (1.25, 2.07)	<0.001	1.36 (1.07, 1.72)	0.011	1.25 (0.95, 1.63)	0.108
Model 2	1.02 (0.92, 1.12)	0.722	1.65 (1.28, 2.13)	<0.001	1.37 (1.08, 1.73)	0.010	1.27 (0.96, 1.68)	0.090
Model 3	1.02 (0.92, 1.12)	0.722	1.61 (1.25, 2.07)	<0.001	1.39 (1.09, 1.77)	0.008	1.26 (0.95, 1.66)	0.109

Model 1: Adjusted for age, sex, BMI; Model 2: Adjusted for age, sex, BMI, fasting glucose, eGFR, current smoking status, current drinking status, and regular physical activity; Model 3: Adjusted for age, sex, BMI, fasting glucose, eGFR, current smoking status, current drinking status, regular physical activity, high animal food intake, and insufficient vegetable intake. Abbreviations: CML, Nε-(Carboxymethyl)lysine; CEL, Nε-(Carboxyethyl)lysine; MG-H1, Nδ-(5-hydro-5-methyl-4-imidazolone-2-yl)-ornithine; IQR, interquartile range; OR, odds ratio; CI, confidence interval.

**Table 3:** Associations of the AGEs’ mixture and subjective sleep disorders in the weighted quantile sum (WQS) analysis.

Outcomes	Direction	WQS Mixture Results			Component Weights (%)	
		OR	95% CI	*p-*Value		
Short sleep duration (<7 h)	Positive	1.16	(0.99, 1.37)	0.065	lnCML	59.58
					lnCEL	38.63
					lnMG-H1	1.79
Poor sleep quality (PSQI > 5)	Positive	1.57	(1.15, 2.15)	0.005	lnCML	53.26
					lnCEL	5.07
					lnMG-H1	41.67
Excessive daytime sleepiness (ESS > 9)	Positive	1.69	(1.13, 2.53)	0.009	lnCML	91.95
					lnCEL	4.25
					lnMG-H1	3.79
Insomnia	Positive	1.93	(1.05, 3.52)	0.027	lnCML	61.41
					lnCEL	32.78
					lnMG-H1	5.82

Models were adjusted for age, sex, BMI, fasting glucose, eGFR, current smoking status, current drinking status, regular physical activity, high animal food intake, and insufficient vegetable intake.

## Data Availability

The data presented in this study are available on reasonable request from the corresponding author (Liegang Liu, lgliu@mails.tjmu.edu.cn) due to confidentiality and privacy considerations.

## Supplementary Materials

The following supporting information can be downloaded at: https://www.mdpi.com/article/10.3390/nu16193282/s1, Table S1: Optimized MRM parameters for UPLC-MS/MS analysis. Table S2: Distributions of plasma AGEs in the study participants. Table S3: Associations of individual AGEs with subjective sleep variables estimated by quantile regression. Table S4: Associations of AGEs’ mixture with subjective sleep disorders estimated by WQS. Table S5: Associations of AGEs’ mixture with subjective sleep disorders estimated by BKMR. Table S6: General characteristics of participants. Table S7: Associations of vegetable and animal food intake and plasma AGEs. Table S8: Associations of dietary macronutrient composition and plasma AGEs. Table S9: Associations between dietary habits and subjective sleep disorders by Chi-square test. Figure S1: Flow diagram of participants. Figure S2: Directed acyclic graph. Figure S3: Spearman rank correlations matrix. Figure S4: RCS curves of individual AGEs and subjective sleep disorders. Figure S5: Subgroup analysis of individual AGEs and subjective sleep disorders. Figure S6: The estimated joint associations of AGEs’ mixture with subjective sleep disorders in BKMR. Figure S7: Associations of individual AGEs and actigraphy-measured sleep variables estimated by quantile regression. Figure S8: The estimated joint associations of AGEs’ mixture with actigraphy-measured sleep variables in BKMR.
