# Supplementary material for: Associations of Advanced Glycation End Products with Sleep Disorders in Chinese Adults
Source: Nutrients. 2024 Sep 27;16(19):3282. doi: 10.3390/nu16193282 (PMC11479084; doi:10.3390/nu16193282)
Supplement: Supplementary file 1 [file nutrients-16-03282-s001.zip › nutrients-3212255-supplementary.pdf]

Associations of Advanced Glycation End Products with Sleep Disorders in Chinese Adults

Linyan Li [1,2], Jianhe Guo [1,2], Xiaoling Liang [1,2], Yue Huang [1,2], Qiang Wang [1,2], Yuxi Luo [1,2], Lei King [1,2], Liangkai Chen [1,2], Xiaolin Peng [3], Hong Yan [2], Ruikun He [4], Jun Wang [5], Xiaobo Peng [1,2] and Liegang Liu [1,2,*]

Supplementary information

Table S1. Optimized MRM parameters for UPLC-MS/MS analysis.

Table S2. Distributions of plasma AGEs in the study participants.

Table S3. Associations of individual AGEs with subjective sleep variables estimated by quantile regression.

Table S4. Associations of AGEs mixture with subjective sleep disorders estimated by WQS.

Table S5. Associations of AGEs mixture with subjective sleep disorders estimated by BKMR.

Table S6. General characteristics of participants.

Table S7. Associations of diet habits and plasma AGEs.

Table S8. Associations of dietary macronutrient composition and plasma AGEs.

Table S9. Associations between dietary habits and subjective sleep disorders by Chi-square test.

Figure S1. Flow diagram of participants.

Figure S2. Directed acyclic graph.

Figure S3. Spearman rank correlations matrix.

Figure S4. RCS curves of individual AGEs and subjective sleep disorders.

Figure S5. Subgroup analysis of individual AGEs and subjective sleep disorders.

Figure S6. The estimated joint associations of AGEs mixture with subjective sleep disorders in BKMR.

Figure S7. Associations of individual AGEs and actigraphy-measured sleep variables estimated by quantile regression.

Figure S8. The estimated joint associations of AGEs mixture with actigraphy-measured sleep variables in BKMR.

Table S1. Optimized MRM parameters for UPLC-MS/MS analysis.

Compound	Ion mode	Mass charse ratio (m/z)	Fragmentor voltage (V)	Collision energy (V)	Collision acceleration voltage (V)	Dwell time (ms)
CML	ESI+	205.1→130.0	84	9	7	30
CML	ESI+	205.1→84.1 [a]	84	17	7	30
CML-d_2	ESI+	207.1→130.0	84	9	7	30
CML-d_2	ESI+	207.1→84.1 [a]	84	17	7	30
CEL	ESI+	219.1→130.0	84	9	7	30
CEL	ESI+	219.1→84.0 [a]	84	17	7	30
CEL-d_4	ESI+	223.1→134.0	102	9	7	30
CEL-d_4	ESI+	223.1→88.1 [a]	102	17	7	30
MG-H1	ESI+	229.1→114.0	102	9	7	30
MG-H1	ESI+	229.1→70.1 [a]	102	25	7	30
MG-H1-d_3	ESI+	232.1→117.0	102	9	7	30
MG-H1-d_3	ESI+	232.1→70.1 [a]	102	25	7	30

Abbreviations: CML, Nε-(Carboxymethyl)lysine; CEL, Nε-(Carboxyethyl)lysine; MG-H1, Nδ-(5-hydro-5-methyl-4-imidazolone-2-yl)-ornithine; ESI+, positive ion mode Electrospray Ionization; MRM, multiple reactions monitoring. [a] Quantitative ion pairs, others are qualitative ion pairs.

Table S2. Distributions of plasma AGEs in the study participants.

Compound (μg/L)	Median	GM	%>LOQ	Percentile							IQR
				Min	5th	25th	50th	75th	95th	Max	
Plasma CML	25.76	31.71	100.00	1.09	10.45	17.13	25.76	60.79	142.23	257.00	43.66
Plasma CEL	13.53	14.17	100.00	4.31	7.12	9.75	13.53	19.34	34.00	78.50	9.59
Plasma MG-H1	79.59	77.55	100.00	3.50	24.50	52.28	79.59	117.14	233.43	370.63	64.86

Abbreviations: GM, geometric mean; LOQ, limit of quantification. LOQ of CML, CEL, and MG-H1 was 0.5, 0.05, and 0.25 μg/L, respectively, and all the inter-assay coefficients of variation and accuracy were within 15%.

Table S3. Associations of individual AGEs with subjective sleep variables estimated by quantile regression.

Exposure	Quantile of outcomes	Sleep duration (min)		PSQI score		ESS score	
		Changes (95% CI)	*P* value	Changes (95% CI)	*P* value	Changes (95% CI)	*P* value
**CML**							
	0.25	-3.58 (-7.90, 0.74)	0.105	0.62 (0.27, 0.97)	0.001	0.48 (0.11, 0.85)	0.011
	0.50	-4.74 (-7.83, -1.65)	0.003	0.41 (0.26, 0.56)	< 0.001	0.61 (0.23, 0.99)	0.002
	0.75	-3.54 (-7.61, 0.53)	0.088	0.51 (0.10, 0.92)	0.016	0.56 (0.15, 0.98)	0.008
**CEL**							
	0.25	-3.77 (-6.71, -0.83)	0.012	0.52 (0.05, 0.98)	0.030	0.74 (0.21, 1.27)	0.006
	0.50	-7.14 (-10.93, -3.36)	< 0.001	0.46 (0.11, 0.82)	0.011	0.57 (0.01, 1.12)	0.046
	0.75	-9.44 (-12.87, -6.00)	< 0.001	0.91 (0.33, 1.48)	0.002	0.75 (-0.25, 1.75)	0.142
**MG-H1**							
	0.25	1.16 (-2.08, 4.39)	0.483	0.77 (0.41, 1.13)	< 0.001	0.32 (-0.11, 0.74)	0.141
	0.50	-1.12 (-4.54, 2.3)	0.522	0.49 (0.20, 0.78)	0.001	0.65 (-0.07, 1.37)	0.077
	0.75	-2.17 (-6.32, 1.98)	0.305	0.66 (0.21, 1.12)	0.004	1.23 (0.52, 1.94)	0.001

Models were adjusted for age, sex, BMI, fasting glucose, eGFR, current smoker (yes, no), current drinker (yes, no), regular physical activity (yes, no), high animal food intake (yes, no), and insufficient vegetable intake (yes, no).

Table S4. Associations of AGEs mixture with subjective sleep disorders estimated by WQS.

Outcomes	Direction	WQS mixture result			Component weights (%)	
		OR	95% CI	*P* value		
Short sleep duration (< 7 h)	Negative	1.03	(0.89, 1.20)	0.653	lnCML	59.58
					lnCEL	38.63
					lnMG-H1	1.79
Poor sleep quality (PSQI > 5)	Negative	1.47	(1.12, 1.92)	0.005	lnCML	3.02
					lnCEL	94.43
					lnMG-H1	2.55
Excessive daytime sleepiness (ESS > 9)	Negative	2.63	(1.57, 4.38)	< 0.001	lnCML	1.20
					lnCEL	54.80
					lnMG-H1	44.00
Insomnia	Negative	1.97	(1.14, 3.42)	0.011	lnCML	6.89
					lnCEL	19.24
					lnMG-H1	73.87

Models were adjusted for age, sex, BMI, fasting glucose, eGFR, current smoker (yes, no), current drinker (yes, no), regular physical activity (yes, no), high animal food intake (yes, no), and insufficient vegetable intake (yes, no).

Table S5. Associations of AGEs mixture with subjective sleep disorders estimated by BKMR.

Quantile of exposure	OR (95%CI)			
	**Short sleep duration (< 7 h)**	**Poor sleep quality (PSQI > 5)**	**Excessive daytime sleepiness (ESS > 9)**	**Insomnia**
0.25	1.03 (0.95, 1.12)	0.80 (0.74, 0.93)	0.91 (0.82, 1.01)	0.92 (0.82, 1.03)
0.30	1.02 (0.96, 1.09)	0.86 (0.79, 0.94)	0.93 (0.85, 1.01)	0.94 (0.86, 1.03)
0.35	1.01 (0.97, 1.06)	0.90 (0.85, 0.95)	0.95 (0.90, 1.00)	0.96 (0.90, 1.01)
0.40	1.01 (0.98, 1.04)	0.94 (0.90, 0.97)	0.96 (0.93, 1.00)	0.97 (0.93, 1.01)
0.45	1.00 (0.99, 1.02)	0.96 (0.94, 0.99)	0.98 (0.96, 1.00)	0.98 (0.96, 1.00)
0.50	1 (ref)	1 (ref)	1 (ref)	1 (ref)
0.55	1.01 (0.99, 1.02)	1.03 (1.02, 1.05)	1.02 (1.00, 1.03)	1.01 (1.00, 1.03)
0.60	1.02 (0.99, 1.05)	1.08 (1.04, 1.11)	1.04 (1.01, 1.08)	1.04 (1.00, 1.07)
0.65	1.04 (0.99, 1.09)	1.12 (1.06, 1.18)	1.07 (1.01, 1.12)	1.06 (1.00, 1.11)
0.70	1.16 (0.99, 1.14)	1.17 (1.09, 1.27)	1.11 (1.03, 1.19)	1.09 (1.01, 1.18)
0.75	1.10 (1.00, 1.20)	1.24 (1.12, 1.38)	1.16 (1.05, 1.28)	1.13 (1.02, 1.26)

Models were adjusted for age, sex, body mass index, fasting glucose, eGFR, current smoker (yes, no), current drinker (yes, no), regular physical activity (yes, no), high animal food intake (yes, no), and insufficient vegetable intake (yes, no). The overall effects on sleep disorders were evaluated by changes in response variables by comparing all AGEs fixed at a certain percentile to the 50th percentile.

Table S6. General characteristics of participants.

Characteristics	All participants (n = 1732)	Subset for sleep quality (n = 383)	Subset for actigraphy (n = 182)
Age, years	51.91 (11.55)	54.99 (9.21)	58.53 (9.46)
Female	999 (57.68)	162 (42.30)	102 (56.04)
Ethnicity/Han	1697 (97.98)	379 (98.96)	180 (98.90)
BMI, kg/m^2	24.55 (3.16)	24.87 (3.17)	24.58 (3.17)
Fasting blood glucose, mmol/L	5.10 (4.71, 5.75)	5.80 (5.20, 6.60)	5.60 (5.00, 6.20)
Current smoker	261 (15.07)	67 (17.49)	25 (13.74)
Current drinker	695 (40.13)	100 (26.11)	39 (21.43)
Physical activity	1262 (72.86)	307 (80.16)	149 (81.87)
Morbidity burden			
None (0 comorbidities)	629 (36.32)	78 (20.37)	44 (24.18)
Moderate (1 - 3)	1033(59.99)	282 (73.63)	129 (70.88)
Significant (4 +)	64 (3.70)	23 (6.01)	9 (4.95)

Unless otherwise noted, data are presented as median (IQR). Abbreviations: BMI, body mass index. Data were presented as n (%) for categorical data, mean (standard deviation) for normally distributed data or median (interquartile range) for non-normally distributed data.

Table S7. Associations of vegetable and animal food intake and plasma AGEs.

	CML	CEL	MG-H1
	Median (IQR)	Median (IQR)	Median (IQR)
Vegetable intake			
Optimal	25.37 (17.00, 59.96)	12.83 (9.53, 18.32)	80.88 (54.10, 120.00)
Insufficient	26.00 (17.37, 61.00)	13.88 (9.94, 19.92)	78.75 (51.50, 115.94)
P value	0.691	0.008	0.107
Animal food intake			
Optimal	26.00 (17.42, 62.89)	13.72 (9.91, 19.50)	79.59 (51.50, 118.91)
High	25.43 (16.50, 54.69)	12.90 (9.33, 18.50)	79.48 (55.00, 112.50)
P value	0.250	0.040	0.687

P value of plasma AGEs among people with different dietary habits was obtained by Wilcxon rank sum test.

Table S8. Associations of dietary macronutrient composition and plasma AGEs.

	CML		CEL		MG-H1	
	**r**	***P* value**	**r**	***P* value**	**r**	***P* value**
Energy (kcal/d)	-0.075	0.539	0.068	0.401	0.058	0.459
Protein (g/d)	-0.129	0.432	0.107	0.241	0.041	0.561
Fat (g/d)	0.031	0.510	0.166	0.017	0.126	0.062
Carbohydrate (g/d)	-0.123	0.163	-0.056	0.398	-0.027	0.706

Spearman correlations between dietary records of a subset with actigraphy data (n = 182).

Table S9. Associations between dietary habits and subjective sleep disorders by Chi-square test.

	Short sleep duration		Poor sleep quality		Excessive daytime sleepiness		Insomnia	
	n (%)	*P* value	n (%)	*P* value	n (%)	*P* value	n (%)	*P* value
Vegetable intake		0.783		0.142		0.950		0.373
Optimal	225 (41.67)		39 (41.94)		18 (19.35)		9 (9.68)	
Insufficient	488 (40.87)		151 (51.71)		54 (18.49)		41 (14.04)	
Animal food intake		0.410		0.394		0.142		
Optimal	547 (40.58)		159 (50.64)		54 (17.20)		45 (14.33)	0.153
High	166 (43.00)		31 (43.66)		18 (25.35)		5 (7.04)	

P value of plasma AGEs among people with different dietary habits was obtained by Chi-square test.

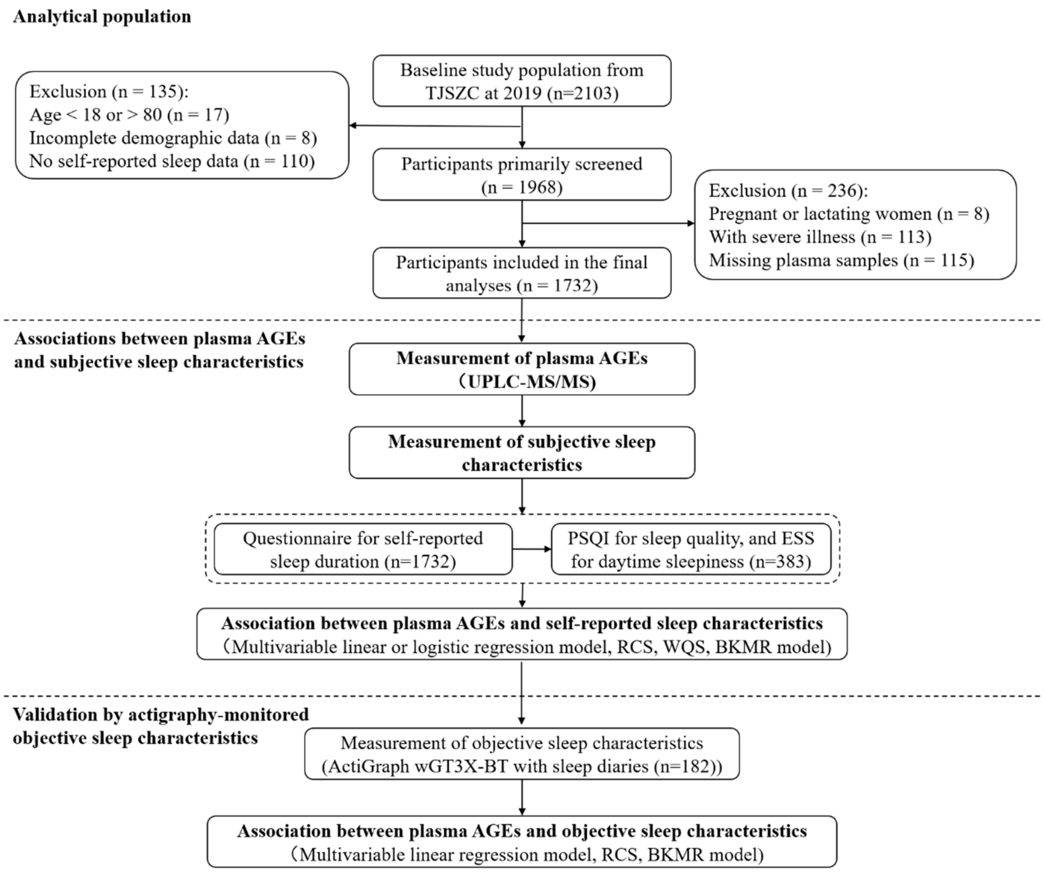

Figure S1. Flow diagram of participants.

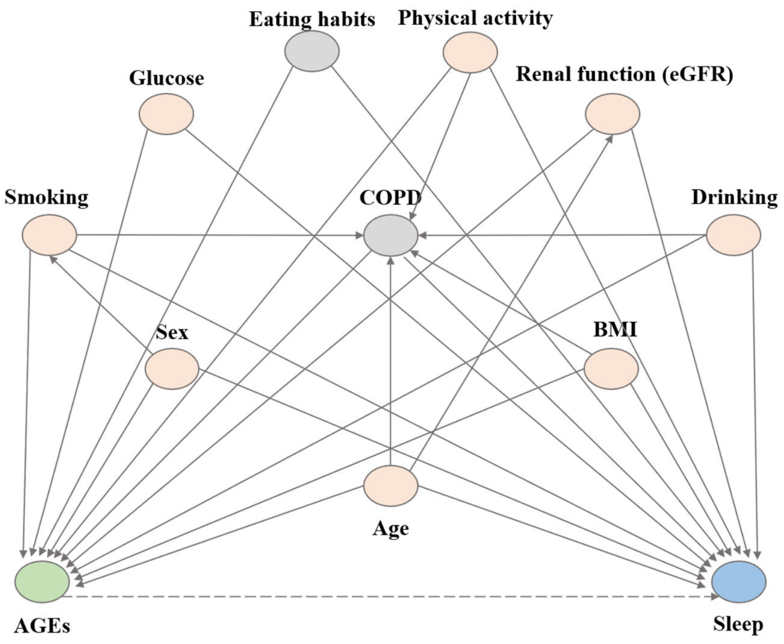

Figure S2. Directed acyclic graph. Light pink circles represent ancestors of the exposure and outcome (ie, confounders), and gray circles represent unobserved (ie, latent) variables. Dotted lines represent causal paths, and gray lines represent biasing paths. The minimally sufficient adjustment set was determined using the DAGitty website. Chronic obstructive pulmonary disease (COPD) was considered to be a collider, which was appropriately accounted for by adjusting for additional variables contained on the backdoor paths shared by this collider. When estimating the association between plasma AGEs and sleep, the final minimally sufficient adjustment set comprised of age, sex, BMI, fasting glucose, eGFR, current smoking status, current drinking status, and physical activity. Adjusting for this set of variables will minimize confounding bias.

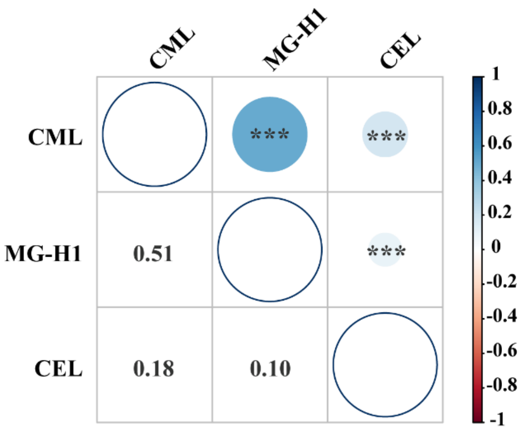

Figure S3. Spearman rank correlations matrix. * $P < 0.05$, ** $P < 0.01$, *** $P < 0.001$.

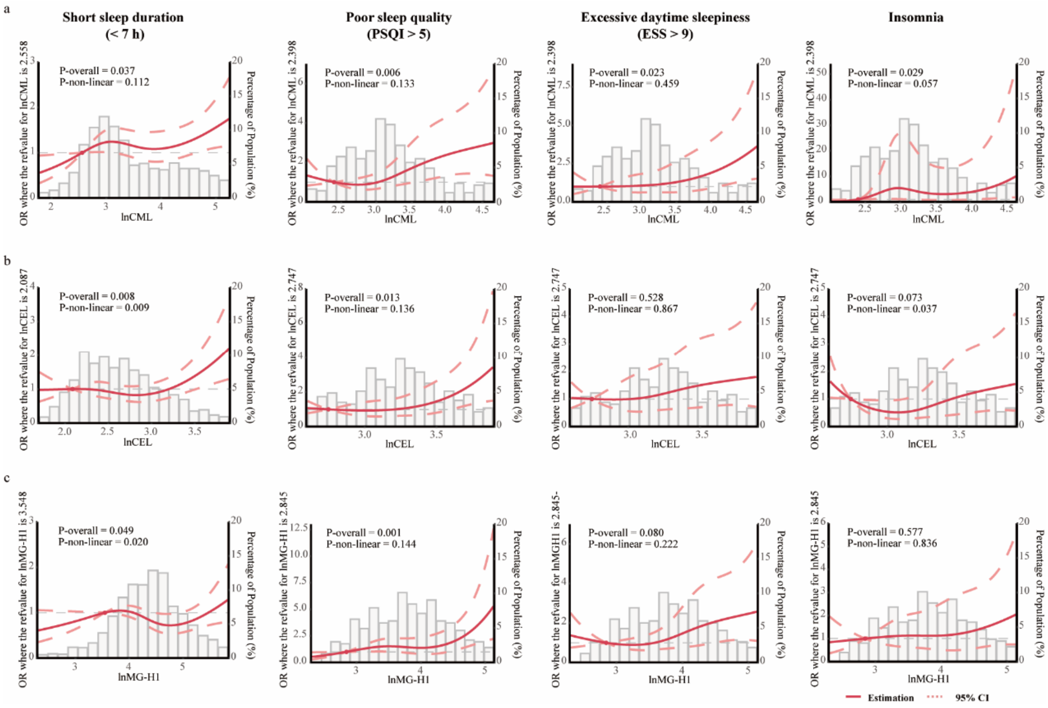

Figure S4. RCS curves of individual AGEs and subjective sleep disorders. (a) lnCML; (b) lnCEL; (c) lnMG-H1. The curb lines represent the estimated β coefficient and the shaded areas represent 95% confidence intervals. The models were adjusted for covariates including age, sex, BMI, fasting glucose, eGFR, current smoking status, current drinking status, regular physical activity, high animal food intake (yes, no), and insufficient vegetable intake (yes, no).

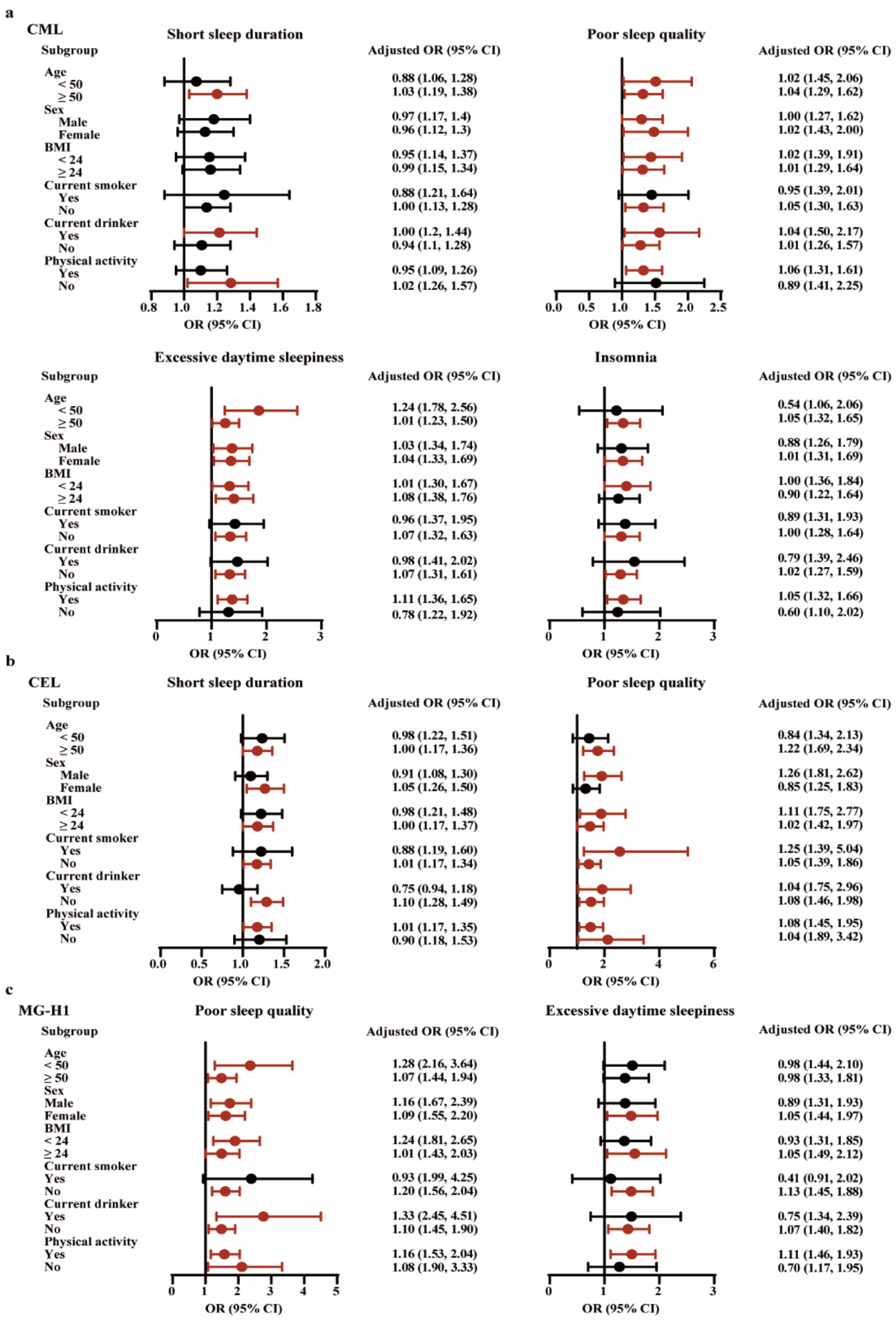

Figure S5. Subgroup analysis of individual AGEs and subjective sleep disorders. The models were adjusted for age, sex, BMI, fasting glucose, eGFR, current smoking status, current drinking status, regular physical activity, high animal food intake, and insufficient vegetable intake, except for the corresponding stratification factor. (a) per IQR increment in CML level; (b) per IQR increment in CEL level; (c) per IQR increment in MG-H1 level.

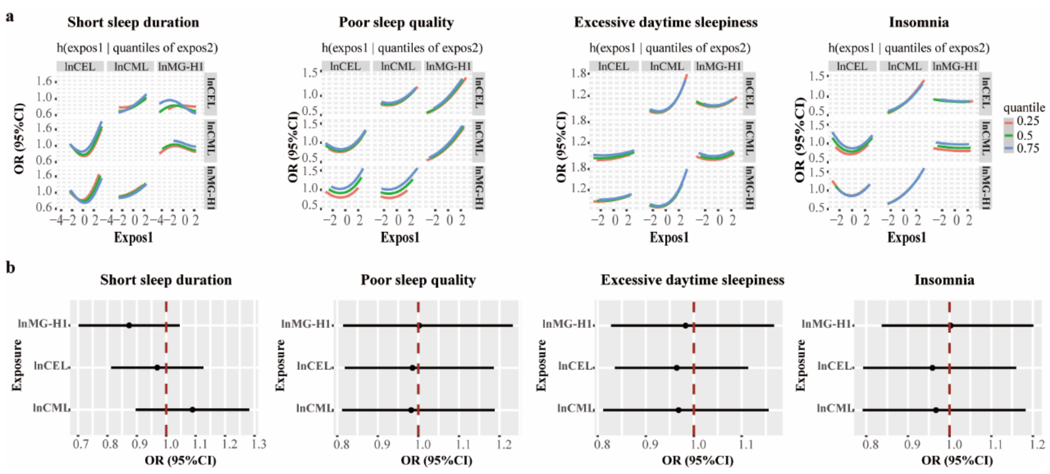

Figure S6. The estimated joint associations of AGEs mixture with subjective sleep disorders in BKMR. (a) Bivariate exposure-response functions for each AGEs and the h(Z) (95% CI) of sleep disorders when one AGEs was fixed at the 25th, 50th, and 75th percentiles and other AGEs were fixed at the median in the BKMR model. (b) Interactive effects of AGEs on sleep variables estimated by BKMR analysis. The models were adjusted for covariates including age, sex, BMI, fasting glucose, eGFR, current smoking status, current drinking status, regular physical activity, high animal food intake, and insufficient vegetable intake.

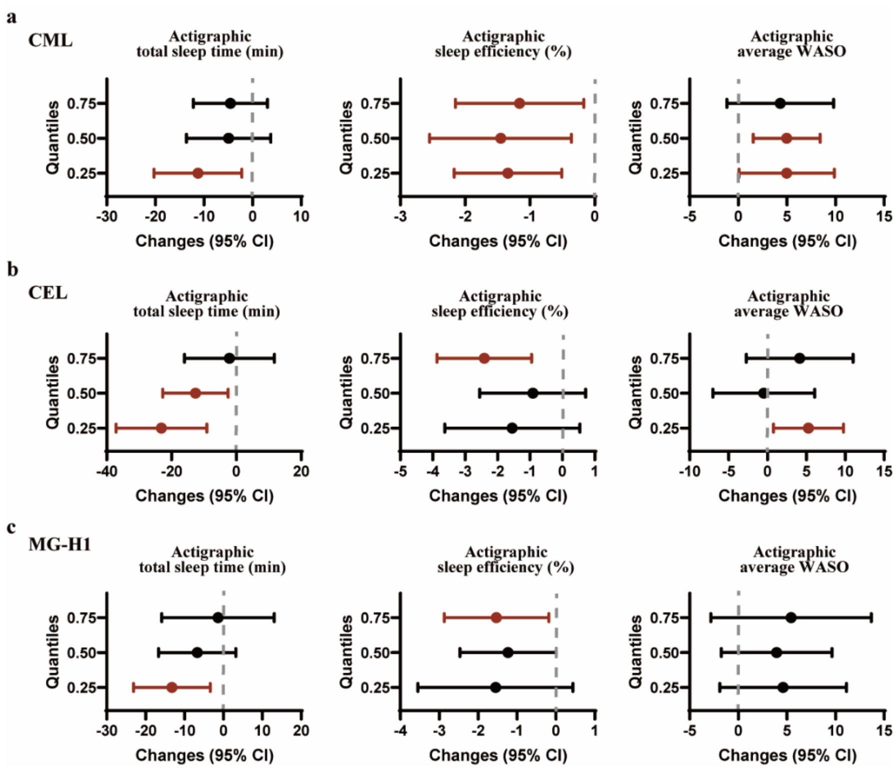

Figure S7. Associations of individual AGEs and actigraphy-measured sleep variables estimated by quantile regression. (a) per IQR increment in CML level; (b) per IQR increment in CEL level; (c) per IQR increment in MG-H1 level. The x-axes depict the changes in objective sleep variables for an IQR increment in exposure, while the error bars represent 95% confidence intervals. The models were adjusted for covariates including age, sex, BMI, fasting glucose, eGFR, current smoking status, current drinking status, regular physical activity, dietary energy, protein intake, fat intake, carbohydrate intake, and tea/coffee consuming status.

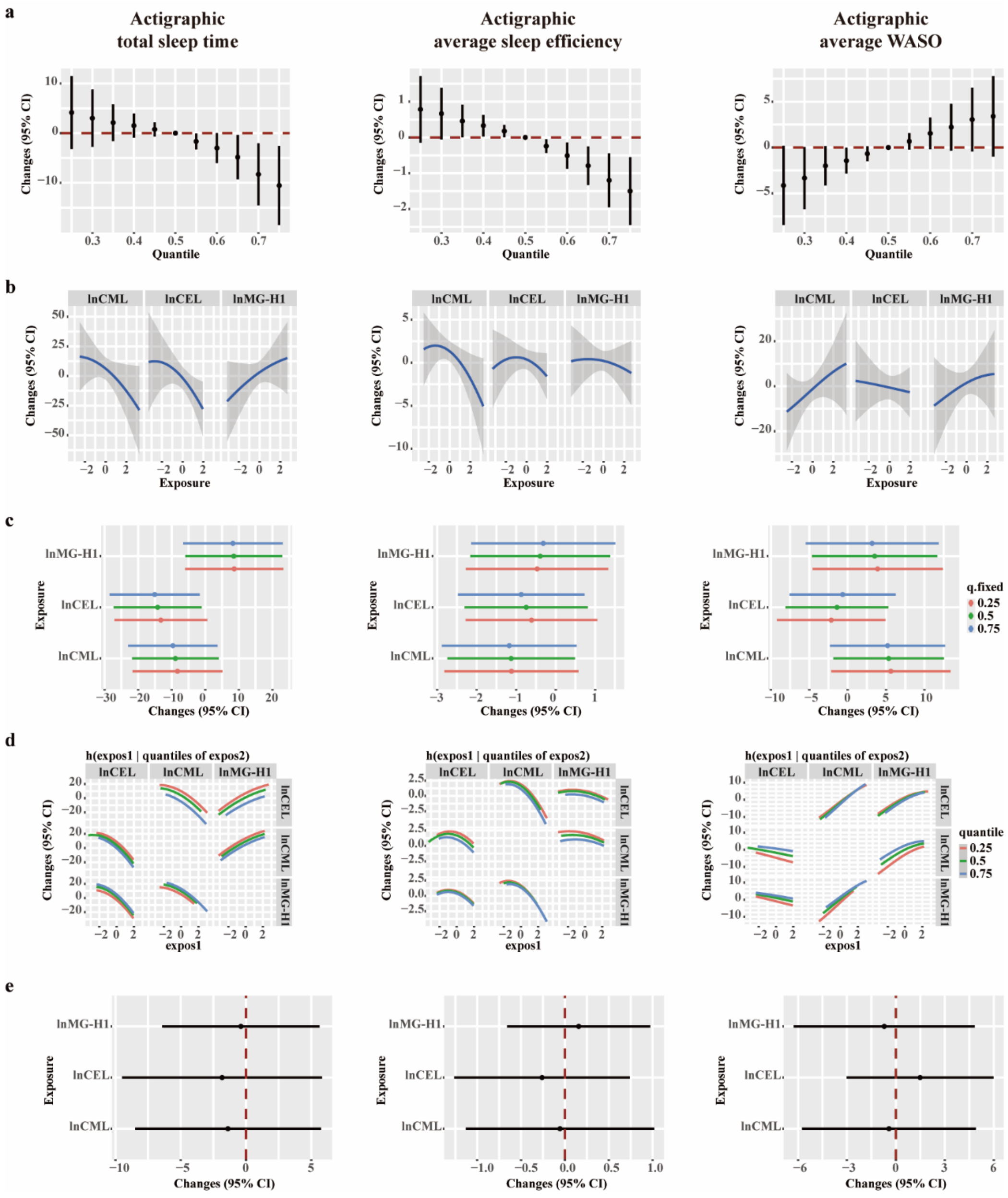

Figure S8. The estimated joint associations of AGEs mixture with actigraphy-measured sleep variables in BKMR. (a) The combined effect of AGEs mixture on sleep at overall concentrations ranged from the 25th to 75th percentiles relative to the median (50th percentile). (b) Univariate exposure-response functions for individual AGEs when the other two were fixed at their 50th percentiles. (c) Estimated changes (95% CI) of actigraphy-measured sleep variables for each AGEs by IQR increment (75th vs. 25th percentile) when all the other AGEs were fixed at the 25th, 50th, and 75th percentiles in

single exposure-response functions in the BKMR model. (d) Bivariate exposure-response functions for each AGEs and the estimated changes (95% CI) of sleep variables when one AGEs was fixed at the 25th, 50th, and 75th percentiles and other AGEs were fixed at the median in the BKMR model. (E) Interactive effects of AGEs on sleep variables estimated by BKMR analysis. The models were adjusted for covariates including age, sex, BMI, fasting glucose, eGFR, current smoking status, current drinking status, regular physical activity, dietary energy, protein intake, fat intake, carbohydrate intake, and tea/coffee consuming status.
